# Visualization of Urban Mobility Data from Intelligent Transportation Systems

**DOI:** 10.3390/s19020332

**Published:** 2019-01-15

**Authors:** Thiago Sobral, Teresa Galvão, José Borges

**Affiliations:** INESC TEC, Faculty of Engineering, University of Porto, 4200-465 Porto, Portugal; tgalvao@fe.up.pt (T.G.); jlborges@fe.up.pt (J.B.)

**Keywords:** data visualization, urban mobility, spatiotemporal data, intelligent transportation sytems

## Abstract

Intelligent Transportation Systems are an important enabler for the smart cities paradigm. Currently, such systems generate massive amounts of granular data that can be analyzed to better understand people’s dynamics. To address the multivariate nature of spatiotemporal urban mobility data, researchers and practitioners have developed an extensive body of research and interactive visualization tools. Data visualization provides multiple perspectives on data and supports the analytical tasks of domain experts. This article surveys related studies to analyze which topics of urban mobility were addressed and their related phenomena, and to identify the adopted visualization techniques and sensors data types. We highlight research opportunities based on our findings.

## 1. Introduction

Urban mobility of citizens can be defined as the whole of trips done with individual and collective modes [1]. The understanding of urban mobility dynamics is a challenge for transportation researchers and stakeholders (domain experts). The proportion of people living in urban areas may rise to figures up to 66% of the world population by 2050 [2]. In various cities, authorities and transportation operators have been investing in Information and Communication Technologies (ICT) to foster digitalization and transforming such environments into smart cities [1]. Intelligent Transportation Systems (ITS) are one example of ICT; they make use of sensors to generate large amounts of spatiotemporal data, which can be analyzed to better understand the travel behavior of citizens in transportation networks. Examples of ITS are Automatic Passenger Counting (APC) systems, inductive-loop traffic detectors, Automatic Number-plate Recognition (ANPR) systems, GPS-enabled devices, e.g., smartphones and Automatic Vehicle Location (AVL) systems. Sensors can be divided into three subgroups: activity-based, device-based and location-based [3]. Activity-based sensors record (derived) information related to an activity performed by an object or entity, e.g., smart card. Device-based sensors continuously record and transmit spatiotemporal and other information, e.g., AVL systems. Location-based sensors record the detection of an entity as soon as it enters within their range, e.g., ANPR systems. ITS generate data with higher granularity, which overcomes the past limitations due to highly aggregate data [4] and poses new challenges regarding data integration, analysis and visualization. It is acknowledged that multidisciplinary approaches are required to implement the smart city paradigm [5], while ensuring data security and privacy [6].

Since the last decade, the development of data visualization systems has gained momentum. They have been applied to the study of various topics in urban mobility dynamics, and are well acknowledged to aid decision making and to detect trends and unusual phenomena in private, public and shared modes of transportation. The inherent multivariate nature of urban mobility data required the development of interactive visualization methods that can effectively answer the analytical tasks of domain experts and provide multiple perspectives on data. Examples of frequently found interaction mechanisms are semantic zoom, brushing and linking [7]. Semantic zoom allows the display of data with various levels of detail as the user zooms in and out. Brushing allows the user to visually select a subset of data with a device, e.g., mouse or touchscreen. Linking provides a coordinated view of a subset of data among multiple visualization techniques. For instance, such mechanism can be found in visualization dashboards.

The earliest applications of visualization systems to urban mobility analysis relied on the capabilities of Geographic Information Systems (GIS) and traditional visualization methods, e.g., bar charts, line plots, and map-based representations in the form of heat maps or choropleths, with limited interaction capabilities. The study of Claramunt et al., published in 2000, is probably the first to stress the need of an architecture for supporting real-time visualization of ITS data within the context of Very Dynamic GIS (VDGIS), a term coined by the authors at that time [8]. In this decade, the advances in computer graphics and its technologies yielded new visualization frameworks, e.g., D3.js [9] and Processing [10], which have been widely used in transportation literature for developing tools that overcome the limitations of GIS software on regards to the visual representation of data. In particular, new visualization frameworks fully support GIS-based file formats such as shape files, which facilitate the geographic representation of an urban network and the spatial events that occur in it. Alternatively, a visualization can feature an urban network as the main subject, and provide new perspectives of a network’s structure. For instance, Agryzkov et al. proposed a graph-based approach for modeling and visualizing an urban network, and found that some classes of graphs are appropriate to identify specific characteristics, e.g., the topology and geometry of the urban layout, and the network morphology [11,12].

### 1.1. Motivation

There are some surveys related to the applications of visualization to transportation and urban mobility, with distinct but complementary views. We clarify the position of this paper on regards to these surveys by summarizing their main contributions.

In 2011, Zhang et al. surveyed the trends related to the evolution of Intelligent Transportation Systems into Data-driven Intelligent Transportation Systems (D^2^ITS), which consist on multifunctional systems driven by algorithms related to computer vision, data visualization, multisource data management and machine learning [13]. Although the study did not solely focus on visualization, it recognized the importance on data visualization for the topic of traffic management. The authors highlighted the need for further visualization research, as the number of surveyed studies in this field was substantially small in comparison to the other ones.

The work of Chen et al. surveyed the visualization of traffic data, with emphasis on the technical aspects of data attributes and their visualization [3]. Many examples were related to the urban mobility domain. The authors grouped the surveyed studies into three high-level categories according to analytical tasks which such studies intended to support: (a) situation-aware exploration and prediction, (b) pattern discovery and clustering, and (c) traffic situations and monitoring. In addition, the data sources found in such studies were grouped into broad categories: trajectory and incident. The study is structured around the following phases of data visualization pipeline: (1) data preprocessing, (2) visual transformation, and (3) visual mapping. In the data preprocessing phases, the authors highlighted the main techniques for data cleaning, matching, organization and aggregation. In the visual transformation and mapping phases, the authors exemplified the different forms of data representation regarding on their attribute types. For instance, the temporal dimension can be represented by radial layouts if the time reference is periodic. Linear time can be statically represented with x–y axes, or dynamically by providing continuous snapshots of data on the visualization canvas, for a given time interval. The spatial dimension was divided into three types: points, lines, and regions. The inherent complexity and amount of point- and line-based data can lead to visual clutter; examples of techniques for overcoming this issue are Kernel Density Estimation (KDE) for both types of data, and edge bundling for line-based data. KDE methods have been used for the generation of heat maps describing entities such as vehicles and pedestrians, as they can smoothen data representation in the visual canvas. Edge bundling summarizes massive graph-based data structures by clustering edges into bundles, at the cost of hindering the identification of flow direction. Region-based data is generally obtained by aggregating point- and line-based data.

A more recent survey by Andrienko et al. [7] covered the aspects of data analysis and visualization that are at the intersection of transportation and movement data, with focus on visual analytics, which leverages the use of algorithms to support analytical reasoning aided by visual interfaces. The authors provided a formal typology of movement data, which allows for the definition of spatial events and trajectories, and in parallel with [3], it also presented several algorithms for data cleaning, organization and transformation, based on such formal typology. The authors grouped the surveyed studies into three broad categories: (a) Movement and Transportation Infrastructure, (b) Movement and People’s Behavior, and (c) Modeling and Planning. The survey also detailed data analysis methods that have been used, e.g., spatial analysis of individual journeys, estimation of origin-destination, and spatial aggregation of trajectories. The authors also identified that studies belonging to category (c) depend on GIS tools.

### 1.2. Contributions

In this paper, we summarize the surveyed studies in two dimensions. The first is related to the topics of urban mobility analysis which have been supported by data visualization methods. We provide a finer classification in terms of analytical tasks, in comparison to [3,7]. The second dimension comprises the type of sensors data which have been used in those studies. We extend the classification used in [3] by defining the ITS types from which data has been retrieved.

This review aims to provide a practical reference to researchers and practitioners. Given a topic on urban mobility analysis, the reader can easily refer to its representative studies. Conversely, given a type of ITS data, it is possible to refer to the studies which have explored it. We argue that our perspective complements existing ones by narrowing the scope of classification of topics on urban mobility analysis, and the data types which have supported them.

### 1.3. Outline

The rest of this paper is structured as follows: Section 2 defines the research questions, and specifies the categorization of surveyed studies. The respective subsections describe each topic and summarize related studies. Section 3 concludes this paper and highlights research opportunities.

## 2. Literature Review

This review aims to answer the following questions:

1. Which phenomena related to transportation have been analyzed using visualizations and which types of data have been exploited?
2. How traditional techniques have been used, and which novel techniques have been proposed?

The figures shown in this chapter illustrate how interactive visualization tools can leverage the combination of multiple visualization techniques, e.g., [14], and extend traditional ones, e.g., [15]. We classified the surveyed studies according to several topics, which will be explained throughout this chapter. For instance, the majority of surveyed studies fall into the following topics of urban mobility analysis:

- Urban traffic flows and monitoring;
- People dynamics in urban environments;
- Road traffic incidents;
- Air pollution.

Based on the surveyed studies, we provide two tables which summarize our findings (see Section 3). In Table 1 we list the surveyed topics and their related studies. Topics were sorted according to the number of related studies. Table 2 provides a classification of data source types which extends the one found in [3], and the studies that used such types.

### 2.1. Urban Traffic Flows and Monitoring

Urban traffic flows analysis and monitoring is the most studied topic in mobility visualization. We begin by mentioning two early works on this subject. Firstly, in 2000, Claramunt et al. stressed the limitations of GIS software for managing data of very dynamic geographic phenomena [8]. Their work proposed a GIS-based prototype for visualizing urban traffic data. Various interactive visualization techniques were used to represent data with different perspectives and levels of aggregation, thus providing a good reference for further works on how different visual perspectives could be useful for domain experts who act on different contexts. For instance, traffic flows could be analyzed (a) spatially, through bidimensional maps, (b) thematically, with area charts, (c) temporally, through line plots that represent time series, and (d) with aggregation, by using bar charts to group intervals of values.

The second historic work, published in 2002, by Shekhar et al, seems to be the first that developed a non-GIS visualization tool for traffic flows data [16]. The CubeView system could be publicly accessed from a web browser, and displayed traffic video, maps with highway traffic intensity and outlier stations for user-specified date and time. Wang proposed the use of three-dimensional (3D) visualizations for a simulation-based traffic impact analysis system [17]. To facilitate interaction with the 3D environment, the visualization interface allows the user to interact with a 2D representation of road network. Through brushing and linking interactions, the selected road segment can be seen on the 3D canvas. 3D visualization of roads and moving vehicles were used by Sewall et al. to represent reconstructed traffic flows from discrete spatiotemporal data [18].

Guo et al. developed TripVista, an innovative visualization tool for analysis of traffic patterns and unusual behaviors at road intersections [19]. The tool tackles the intrinsic difficulty due to multidimensional nature of such data by introducing several visualization techniques. Geographic visualization is combined with abstract representations such as parallel coordinate plots, which have been used to represent multidimensional data, and scatterplots. The user can interact simultaneously with all visualization techniques through brushing and linking. The system also makes use of the ThemeRiver visualization technique, which is used to depict thematic variations over time [20]. The authors stated that the feedback was positive among domain experts.

**Figure 1:**
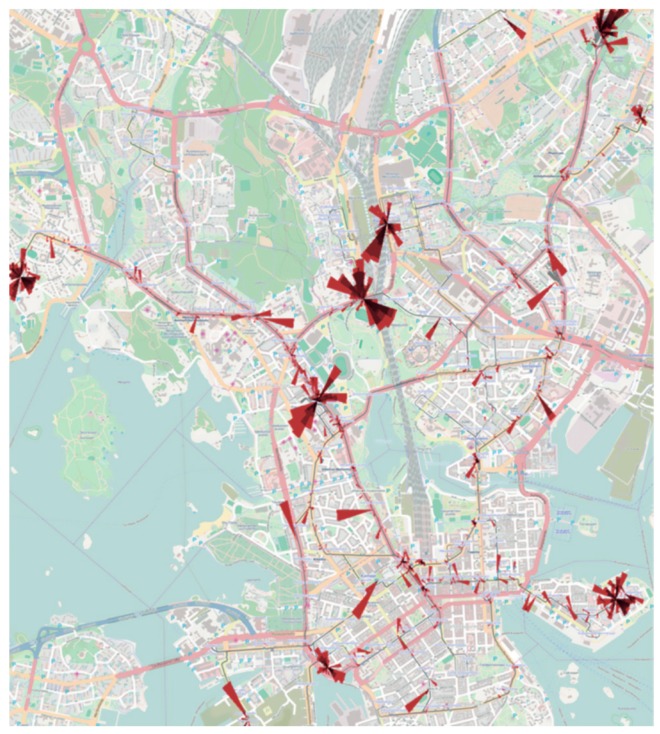
Rose diagrams applied to the spatiotemporal analysis of traffic congestion in Helsinki [15]. The transparency of each circular segment is used to depict the number of occurring traffic jams. The size of each circular segment represents the duration of traffic jams.Rose diagrams applied to stops and congestion analysis

Andrienko et al. [15] proposed the application of rose diagrams to spatiotemporal analysis of traffic congestion (see Figure 1). In accordance to the original visual metaphor, circle segments represent time (hours of the day). The transparency of each circular segment is used to depict the number of occurring traffic jams. Finally, the size of each circular segment represents the duration of traffic jams. The authors tested the visualization technique with public transportation system data from Helsinki, Finland. In Figure 1, each rose diagram is centered on a city landmark.

**Figure 2:**
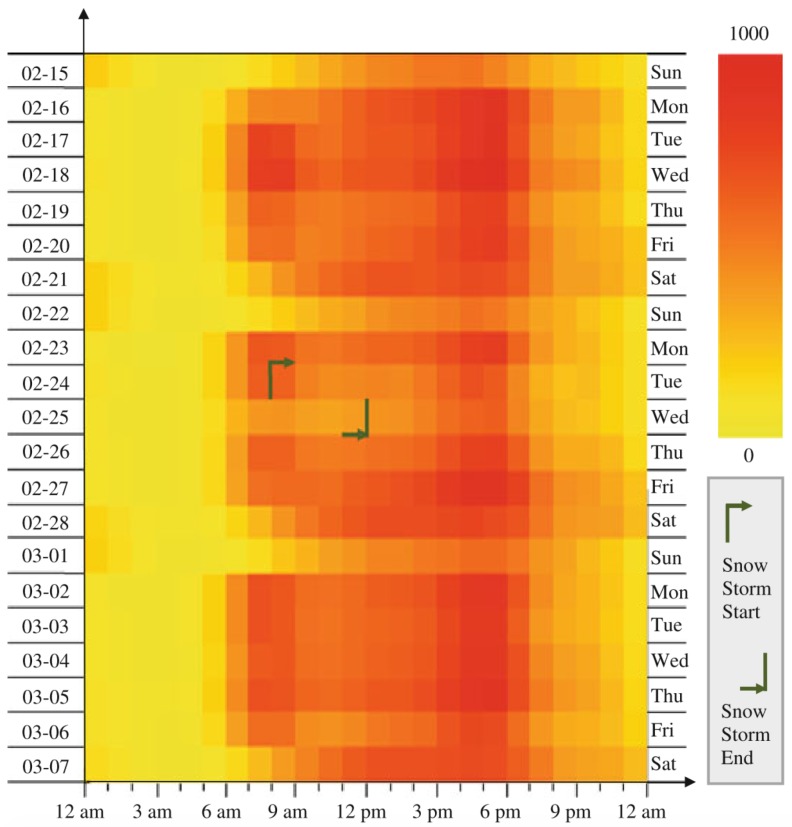
A heat map matrix visualization for traffic congestion analysis [21].A heat map matrix visualization for traffic congestion analysis

Some authors focused on the temporal perspective of traffic flow analysis [21,22,23,24] using abstract visualization techniques. Song and Miller proposed a heat map matrix to analyze congestion patterns across two temporal granularities: days of the weeks or months, and time of the day [21], as shown in Figure 2. Such matrices can be effective on identification of abnormal patterns and have been applied to other visualization tools [25]. Liu et al. and Pu et al. applied circular heat maps for the same purpose [22,23], which were overlaid on a map.

The work of Chen et al. [24] highlights the importance of the semantic zoom interaction for analyzing phenomena at different levels. Speed bottlenecks retrieved from vehicle sensors can be analyzed in various temporal granularities in a heat map matrix. As the user selects the desired time period, the visualization technique adapts to show the respective vehicle speeds and flow intensity.

**Figure 3:**
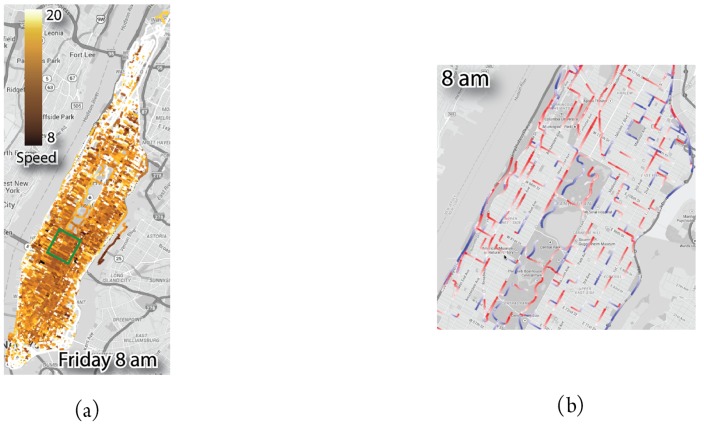
Other forms of representing heat maps on geographic maps. (**a**) consists of a heat map overlay, while (**b**) provides colors to road segments according to a given scale [27].

Other ways of depicting traffic flows using map-based techniques are overlaying heat maps on geographic maps [25,26,27,28,29,30] (see Figure 3a) or road segments (pathlines) [25,27,29,30] (see Figure 3b), which can aid the detection of phenomena such as traffic jams.

**Figure 4:**
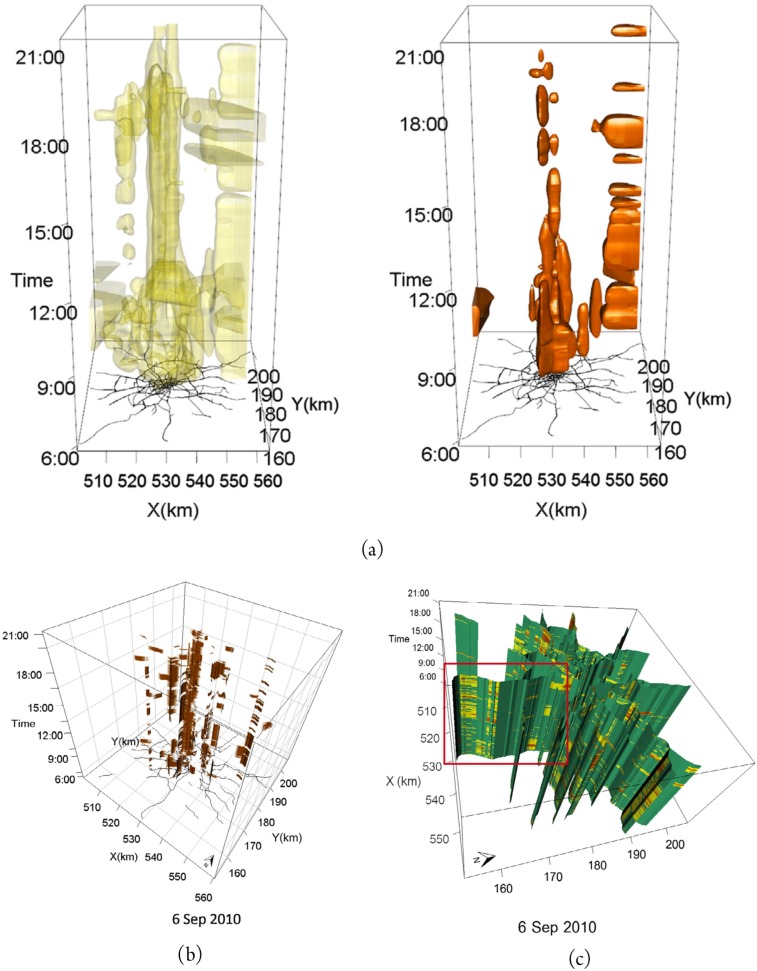
The isosurface (**a**), network-constrained isosurface (**b**), and the wall map (**c**) visualization techniques for exploring congestion evolution [31].

Cheng et al. revisited the space-time cube proposed by Hägerstrand and applied three 3D visualization techniques to the exploration of congestion patterns: isosurface, network-constrained isosurface, and wall map [31]. The authors applied those techniques to a dataset containing vehicle traffic data from London, extracted from ANPR systems. An isosurface shows points of equal value on a 3D shape (Figure 4a). The network-constrained isosurface enhances the accuracy of the former, as it assumes that congestion values will be interpolated between roads, i.e., where there are no cars (Figure 4b). A shortcoming of both methods is that they become less effective on analyzing particular road links. The wall map overcomes such limitations by reducing visual clutter and revealing congestion levels on road links (Figure 4c).

Tanaka et al. combined map-based visualization and traditional techniques on a geospatial dashboard for winter road management using vehicle sensor and microblogging data [32]. The dashboard provides coordinated multiple views, through brush and linking interactions, and features traditional visualization techniques such as bar charts, histograms, scatterplots and dendograms. The tool can be manipulated on touch-screen-enabled devices. Wang et al. also proposed a dashboard for exploring real traffic situations, and features a map-based visualization technique overlaid with heat maps, with bar charts, histograms and scatterplots [33].

Hsieh et al. approached the problem of traffic flow analysis using video stream data [34]. The visualization system uses video streams from one location to depict traffic situation of other places.

Huang et al. proposed the TrajGraph system to analyze traffic flows using taxi trajectories data [35]. The interface provides multiple coordinate views with different visualization techniques. A map-based view was combined with a rose diagram overlay that was used to represent traffic information and network centralities. Line plots and an abstract, graph-based representation of the road network were used.

**Figure 5:**
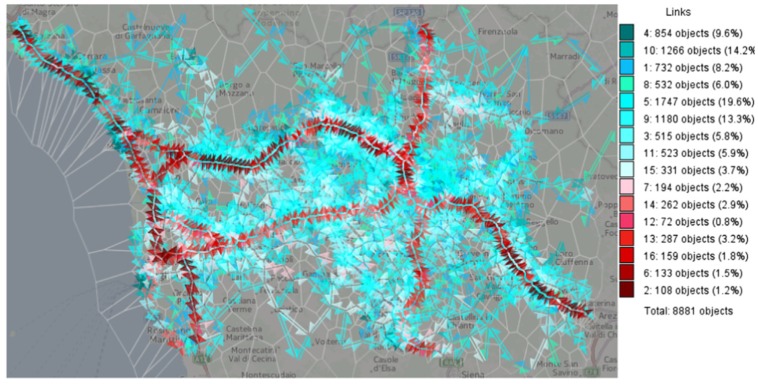
Flow map visualization color-coded in terms of mean speed in road links [36].

Clustering techniques can be combined with categorical color scales to depict cluster membership. For instance, Andrienko et al. proposed a flow map visualization in which colors are given according to the cluster membership of the mean speeds on road links [36], as shown in Figure 5.

### 2.2. People Dynamics in Urban Environments

The study of people dynamics has been mostly focused on detecting urban hotspots. A major data source that supports related works consist of mobile phone data [37,38,39,40,41,42]. Other data sources were socio–economic data [37], taxi GPS trajectories [43], travel diary survey data [44], model-generated OD matrices [44], vehicle sensor data [36], and microblogging data [14,42].

**Figure 6:**
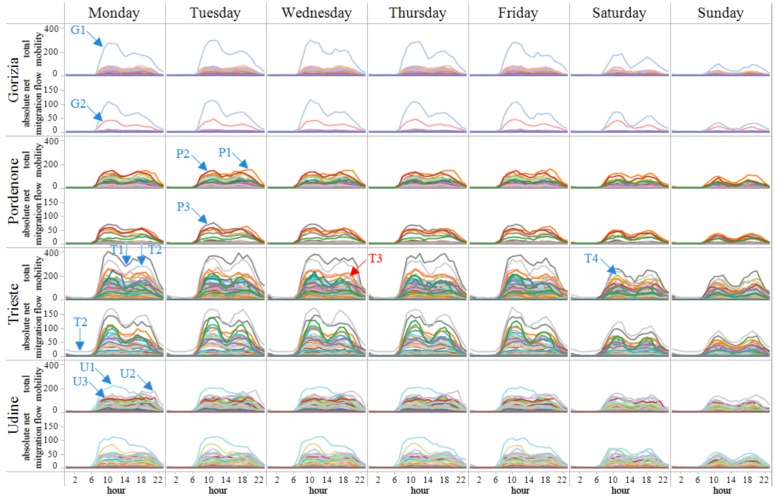
Sparklines visualization of temporal variation of total mobility and net migration flow on each urban center [38].

Kang et al. used the space-time cube visualization to analyze aggregate mobility dynamics of people in urban settings [37]. Sagl et al. used 2D map-based and abstract visualizations for exploring mobility patterns in four Italian cities [38]. Map-based heat maps were used to estimate the spatial density of total mobility. Sparklines (see Figure 6) were used to analyze the temporal variation of total mobility and net migration flow on each urban center. Map-based heat maps and sparklines were used by Zuo and Zhang for the same purpose [39].

**Figure 7:**
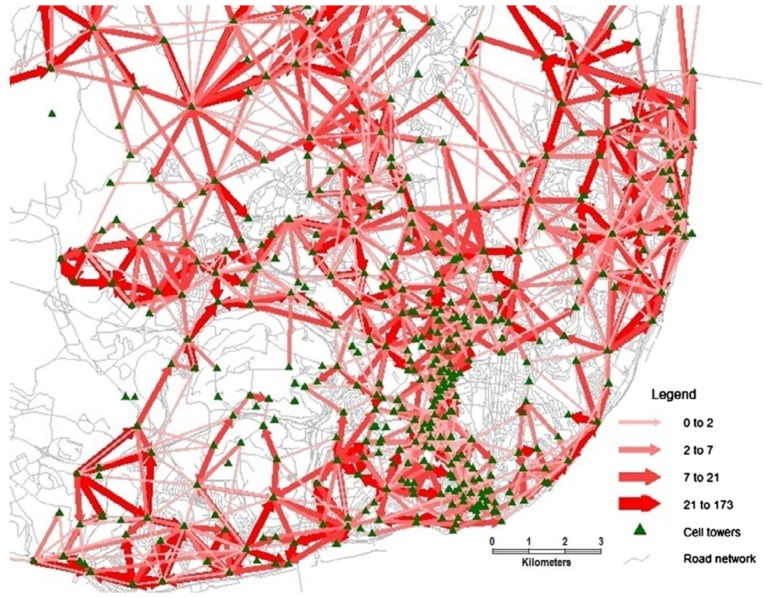
Map-based visualization of handover flows using flow maps [40].

**Figure 8:**
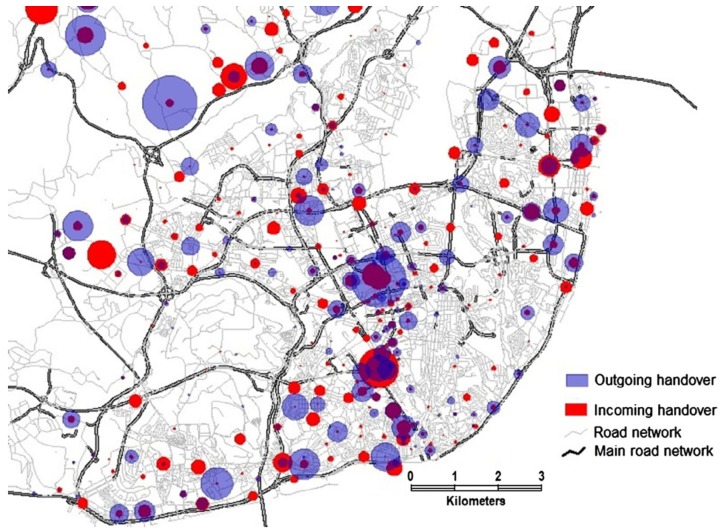
Map-based visualization of volume of incoming versus outgoing handover flows using sized circles [40].

Demissie et al. analyzed cellular network handover information to test certain assumptions of mobility patterns in Lisbon, Portugal [40]. Visual exploration of data consisted of map-based visualizations such as flow maps (Figure 7) and sized circles (Figure 8). The former was used to depict the direction and strength of the handover flow. The latter provided an effective comparison between incoming and outgoing handover on main road links.

**Figure 9:**
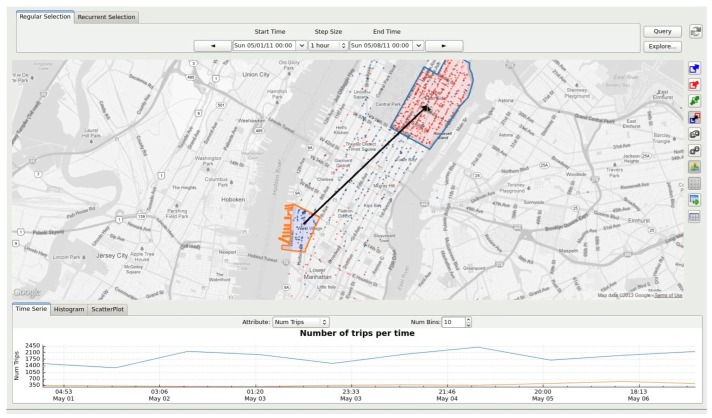
The TaxiVis system for exploring mobility patterns from taxi trips [43] (adapted from [45]).

Ferreira et al. proposed the TaxiVis system for exploratory visualization of taxi trips, using the city of New York, NY, USA, as a case study [43]. The system provides multiple coordinated visualizations. For instance, map-based choroplets and heat maps are used for analyzing trip density within city regions. Line plots are combined with scatterplots and bar charts for visualizing temporal and thematic information, such as trip duration, fare amount and distance. Figure 9 shows the main system interface.

**Figure 10:**
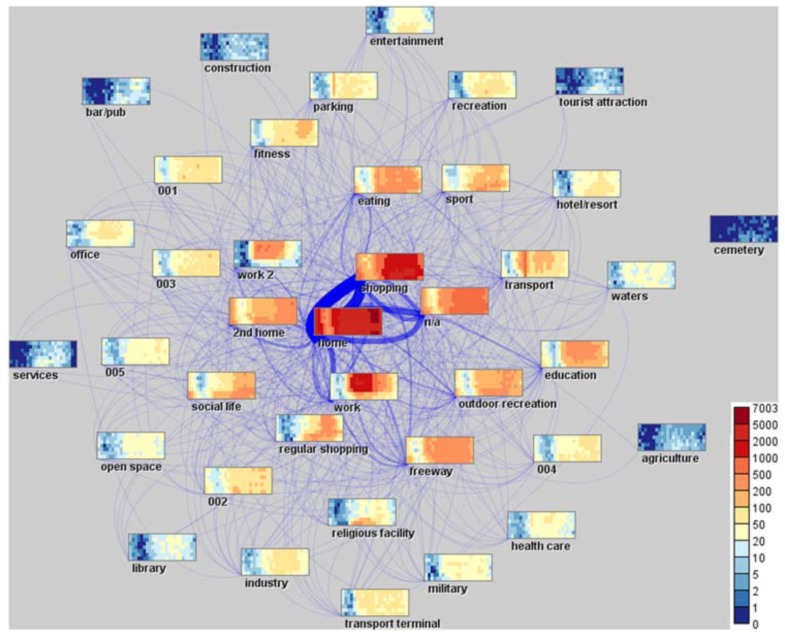
Semantic space map for visualization of mobility flows [41].

Andrienko et al. developed a visual analytics system for supporting mobility analysis from episodic data, while preserving citizen’s privacy [41]. Episodic data was retrieved for each individual, from which an algorithm was used to derive the most likely meaning of the places visited by users. Figure 10 shows an example of the semantic space map visualization technique, which represents flows between several place categories, combined with heat map matrices for each of those places. The widths and opacity of lines are proportional to the total moves between each origin-destination pair. The color scale corresponds to temporal clusters of similar flows.

Von Landesberger et al. tackled the issue of visual clutter that may occur in flow maps [42]. By introducing spatial and temporal simplifications through cluster analysis, graph-based flow maps were combined with temporal cluster representations to give insights on regular daily and weekly patterns of the population.

**Figure 11:**
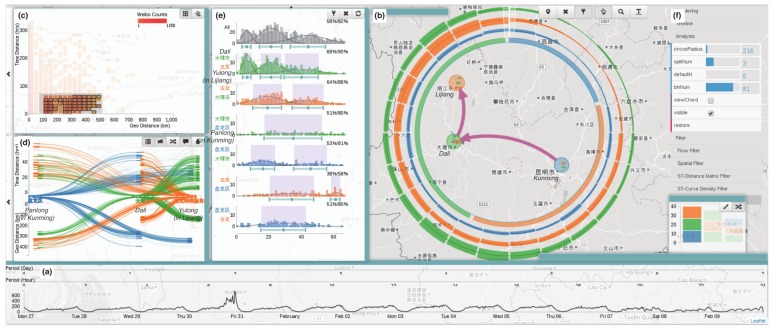
A visual analytics system for exploring sparse microblogging data. Several visualization techniques are interrelated [14] (extracted from [46]). Time plots (**a**) and heat map matrices (**c**) represent the distribution of movement in distance and time. The map-based visualization in (**b**) displays aggregate spatiotemporal flows between cities. Finally, (**d**) features Sankey diagrams to represent pairwise movements in time.

Chen et al. proposed a visual analytics approach to address the shortcoming of microblogging data, which is typically sparse [14]. The system features several abstract visualization techniques, connected with brushing and linking interactions, which are combined with a map-based visualization for displaying aggregate spatiotemporal data (Figure 11b). The work makes a novel use of Sankey diagrams (Figure 11d) to represent pairwise movements in time. Heat map matrices (Figure 11c) and time plots (Figure 11a) were used to represent the distribution of movement in distance and time, and for temporal data filtering, respectively.

Nunes et al. [47] developed the Beanstalk platform for analysis of tourism dynamics, such as trip itineraries, based on passenger counts retrieved from activity-based data, e.g., passenger count from points of interest, and survey data retrieved from tourism authorities. The platform contains various types of interactive visualization techniques. Chord diagrams are used to display movement information between points of interest. Time-based occupancy rates in points of interest are displayed on a heat map matrix.

### 2.3. Road Traffic Incidents

Visualization of road traffic incidents has been supported by datasets related to car incident records [48,49,50,51,52], and vehicle sensor data [52].

Li et al. used a 3D GIS-based visualization to represent potential crash risks on road links, by ranking and estimating segments with potential for vehicle crashes [48]. A 3D map with the road segments is overlaid with bar charts. The height of each bar represents the crash risk of a given location. Pack et al. proposed a visualization tool that combines multiple coordinated views [49]. A map-based visualization was used to display the location of each accident. A bar chart histogram showed the frequency of each accident property, e.g., fatality, injury, roadwork. Given that an accident can be related to multiple properties, parallel coordinate plots were used to explore the relationship between each property. Finally, scatterplots and heat map matrices allow exploring the pairwise relationship between variables. Map-based heat maps have also been used to represent vehicle incidents [50,51]. Plug et al. evaluated the effectiveness of heat maps with domain experts and general public, and reported positive results [51].

Anwar et al. proposed a new map-based visualization technique for exploration of road conditions under traffic incident conditions [52]. The Traffic Origins visualization shows a red circle glyph whenever an accident occurs, and displays the road conditions in the surroundings of the accident location. After the accident, the glyph changes its color to represent the road conditions after the accident, e.g., heavy traffic or breakdowns.

### 2.4. Air Pollution

Visualization of air pollution uses data from vehicle sensors [53] and model-based estimations of emissions, dispersions or heat [17,53,54], model-based traffic flow data [17], bus AVL data and GPS trajectories [55], and video streams [56].

All surveyed studies used map-based visualizations with heat map based overlays. Rebolj et al. and Wang et al. used GIS and 3D maps in combination with bar charts to identify road links with high air pollution levels [17,53]. Li et al. proposed a web-based visualization system for visualizing emissions of diesel buses on a microscopic scale, i.e., bus route segments [55]. Heat maps are applied to road segments to indicate the emissions rate along several bus routes. Morris et al. used video stream data to estimate traffic flows and emissions on highway segments [56]. The authors used line plots to depict the evolution of emissions of pollutants over time.

**Figure 12:**
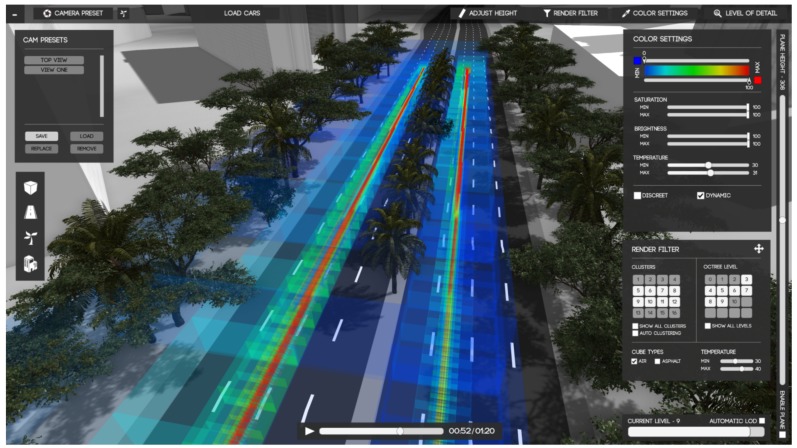
CityHeat visualization tool for microscopic simulation and analysis of traffic heat on a three-dimensional virtual city environment [54] (extracted from [57]).

Cristie et al. proposed an interactive visualization tool, CityHeat, for cellular automata based simulation and analysis of traffic heat in microscopic scale [54]. The tool, as shown in Figure 12 provides interaction tasks such as pan and zoom, filtering, and temporal querying. Heat cubes represent the temperatures of road sections according to simulated traffic intensity and vehicle types.

### 2.5. Travel Behavior on Public Transportation Systems

Data for visualization of travel behavior on public transportation systems (PTS) was retrieved from smart cards (AFC) [58,59,60,61], traveler information systems and vehicle sensors [62].

Fuse et al. used smart card data from a Japanese city to analyze travel behavior under certain weather conditions [58]. Line plots and bar charts were effectively used simultaneously to analyze passenger ridership and precipitation amount. Aggregate time series data was represented using stacked bar charts for analysis of public transportation use frequency under different weather conditions, e.g., sunny or rainy, and day type, e.g., weekday or holidays. Roux et al. implemented map-based heat maps to analyze passenger flows [59].

**Figure 13:**
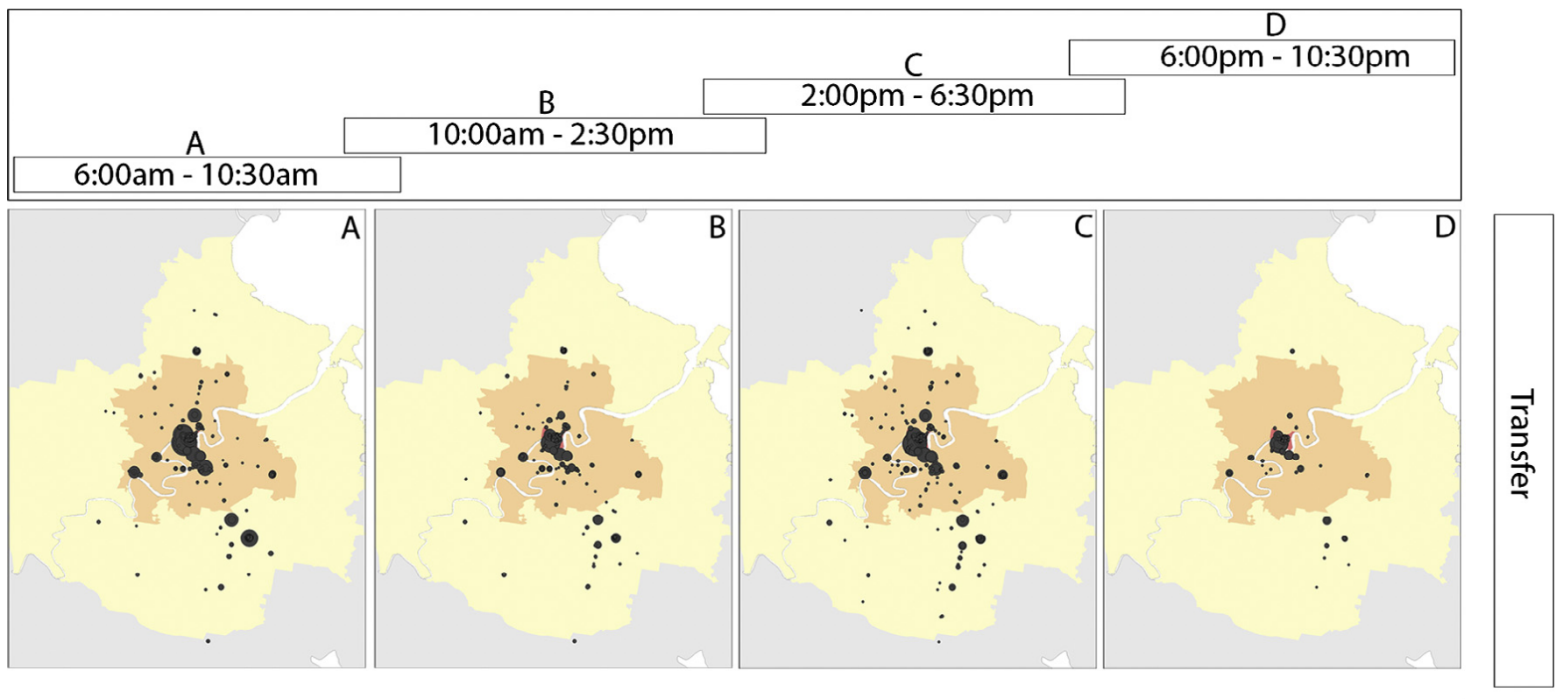
The flow-comap visualization technique for exploring spatiotemporal mobility patterns [60].

Tao et al. introduced the use of flow-comaps to visualize aggregate flow patterns of passengers at a network level [60]. Such technique proved to be useful to identify the major flows of bus passengers over a time period. Flow-comaps combine flow maps with conditional plots (see Figure 13). Once again, map-based heat maps and line plots were identified for spatial and temporal analysis of passenger flow patterns, respectively.

Zeng et al. stated that visualization techniques tend to focus on the network topology across stops, ignoring mobility factors such as riding and waiting times. They proposed three visualization techniques for tackling this gap, focusing on a variety of time-related factors that impact mobility in PTS [61]. Two techniques are discussed in the following paragraphs.

**Figure 14:**
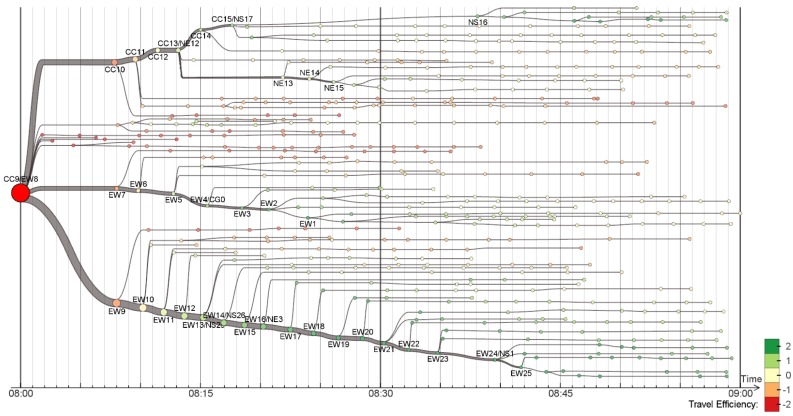
The isotime visualization technique for exploration of time efficiency of journeys based on a starting stop [61] (extracted from [63]).

**Figure 15:**
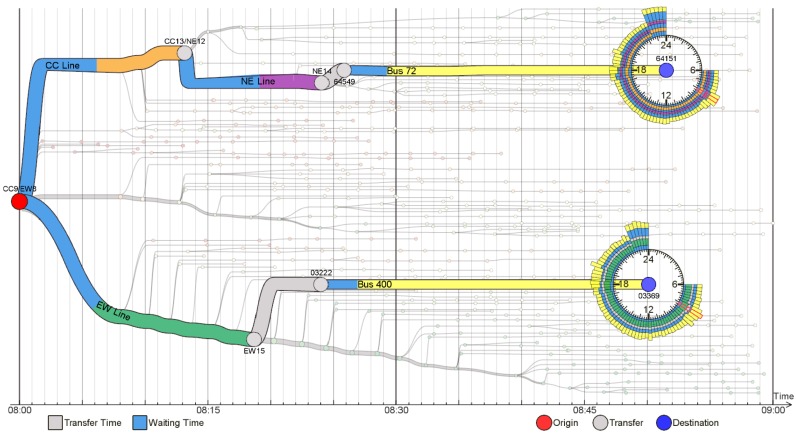
The OD-pair journey view uses the isotime visualization technique to analyze different route options for the same OD-pair [61] (extracted from [63]).

Figure 14 exemplifies the isotime flow map, which linearizes a flow map in a parallel isotime. It is possible to visualize the time efficiency of journeys that start at a certain stop (red circle on the left side of the picture), which is calculated in terms of standard deviations of the mean travel time. Each small node corresponds to a bus stop. The OD-pair journey view (see Figure 15) is based on the isotime visualization technique. Given an origin and destination, it is possible to visualize the transfer and waiting times, as well as round-the-clock variations with the mobility wheel glyph, which is used to encode such temporal information.

### 2.6. Level of Service on Public Transportation Systems

Visualization of level of service on public transportation systems uses data from transit reports [64], tram AVL data [65,66], subway AVL and schedule data [67].

Yu et al. used GIS-based map visualization to analyze bus schedule adherence, comparing static and realtime data for a set of stops [64]. Currie et al. and Mesbah et al. proposed a methodology for mining tram AVL data to support reliability analysis (actual versus scheduled travel times), and trend analysis of reliability [65,66]. The resulting data was visualized with geographic heat maps.

**Figure 16:**
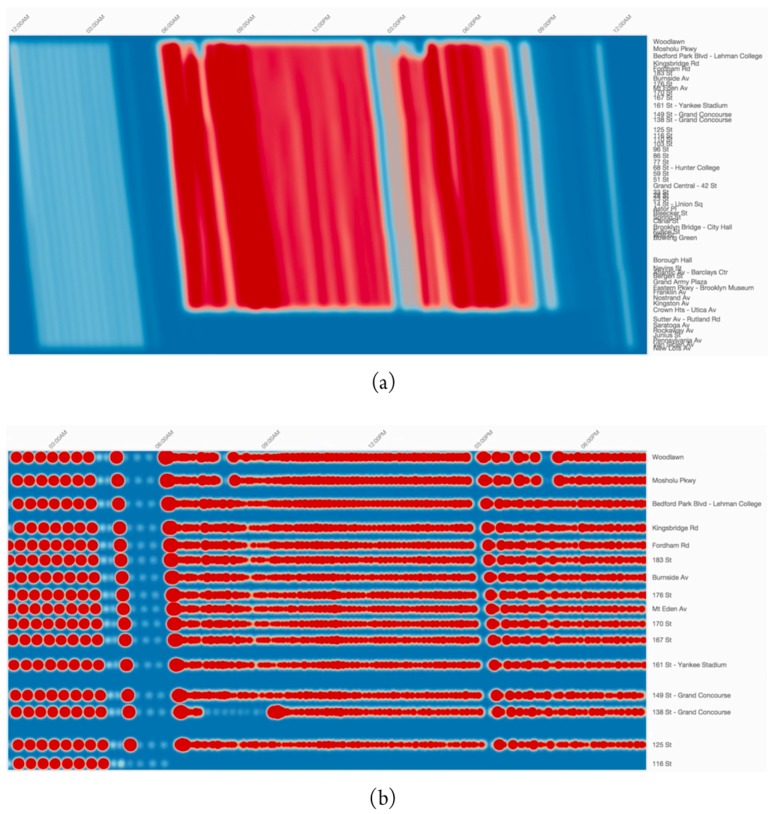
The Trips Explorer (TR-EX) online visualization tool for exploration of transportation schedules at trips and stops levels. Trips visualization is shown on (**a**), while stops visualization is shown on (**b**) with a different time window and reduced number of stops [67] (adapted from [68]).

Palomo et al. proposed an online visualization tool, Trips Explorer (TR-EX), for analyzing reliability of transportation schedules [67]. The tool uses kernel density estimation techniques, and allows users to compare planned timetables against real service, to analyze speed profile at route segments level, and to assess delay, wait time and reliability at station level. Figure 16a shows the visualization of frequency for inbound trips of a subway line of New York City. Figure 16b shows the visualization at stops level with another time window for the same line. Warmer colors indicate higher average delay.

### 2.7. Trip Patterns

The few studies regarding analysis and visualization of trip patterns make use of taxi trajectory data, hence all of them are related to taxi trips [43,69,70,71]. The TaxiVis visualization system of Ferreira et al. has already been featured in the topic People Dynamics in Urban Environments.

Liu et al. used geographic heat maps to analyze the spatial distribution of pick up and drop off points [69]. Mao et al. used GIS-based visualizations to analyze spatiotemporal trip patterns [71]. Map-based techniques such as choroplets and flow maps were used to analyze travel density and connectivity.

**Figure 17:**
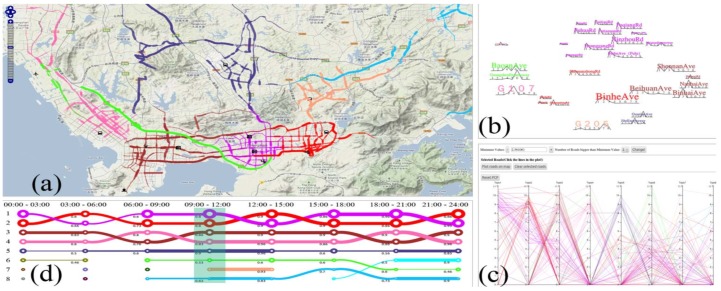
Multiple coordinated visualizations for analyzing hidden themes of taxi trip patterns, which are defined by the authors as “taxi topics”. In (**a**), taxi topics are color coded and represented on a map. (**b**) Uses word clouds to depict street representativeness for each topic. The parallel coordinates technique in (**c**) displays the relationship between topics. Finally, (**d**) depicts the temporal relationship between topics in a timeline [70] (extracted from [72]).

Chu et al. proposed a novel approach to trip patterns analysis [70]. Spatiotemporal information is transformed into contextual semantic information, which is used to drive hidden themes, named as taxi topics by the authors. Each topic, generally the name of a street or avenue, is related to a certain pattern. The visualization system that supports such analysis provides multiple coordinated visualization techniques, as in Figure 17. In (a), topics are represented on a map. Word clouds featuring street names are used in (b) along with sparklines to depict the representativeness of each street on a topic. Parallel coordinates view (c) is used to explore the relationship between topics. Temporal relationship between topics can be explored in (d).

### 2.8. Other Topics

In this section we discuss topics for which we have found three or less studies. Some of them may suggest future exploration by other researchers.

Some works proposed the concept of big city data, i.e., data from several systems for the purpose of gaining an holistic perspective of the dynamics of a city. In 2012, Corral-Soto proposed the 3DTown system for real-time integration and visualization of 3D urban models, video streams, sensors and several real-time information sources [73]. Visualization techniques are mostly GIS-based to depict building and vehicle 3D models. Pedestrian tracking is also represented on maps using 2D glyphs and heat maps for analyzing pedestrian density. Lv et al. and Li et al. proposed a web browser-based virtual reality GIS focused on 3D visualization of city dynamics [74,75]. 3D building models are also used to facilitate the identification of the main city points, although several visualization techniques were combined for displaying different types of data. For instance, passenger flows on PTS stops were represented with 3D bar charts and overlaid 2D heat maps. Video stream data was also overlaid on the 3D map. Bar and pie charts were used to visualize socio–economic information. Line plots were used to show temporal information about passenger flows.

**Figure 18:**
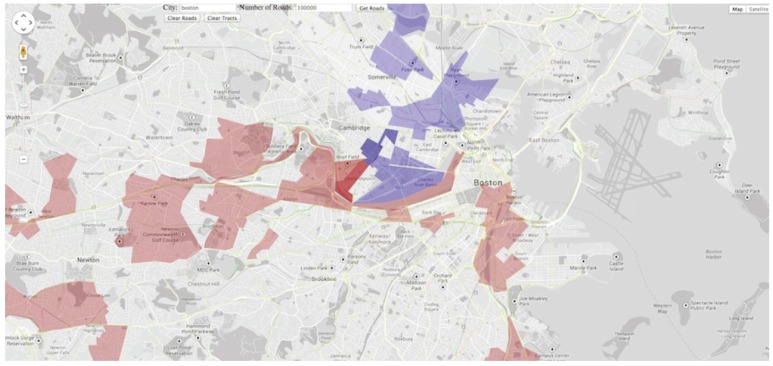
Map-based visualization of travel demand. City regions that attract trips are encoded in blue. Regions that generate trips are encoded in red [76].

Visualization of travel demand used data from mobile phone records [76], socio–economic and travel survey records [76], and taxi GPS trajectories [77,78]. Toole et al. proposed a model for travel demand estimation and proposed an interactive visualization tool for engaging transportation stakeholders [76]. The tool shown in Figure 18 uses a map-based visualization for characterizing city regions that are attract (blue) and generate (red) trips.

**Figure 19:**
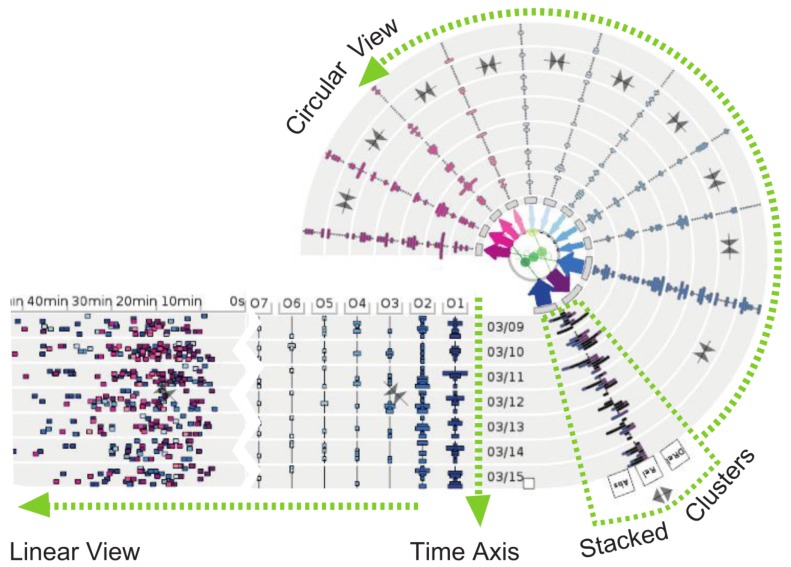
The OD-Wheeltechnique for exploring origin-destination patterns from trajectory data [78].

Lu et al. proposed a novel visualization technique for exploring origin-destination patterns [77,78], which was positively evaluated by domain experts. The technique was evaluated with taxi trajectory data, although it can be used for general trajectory data. The OD-Wheel (see Figure 19) features a linear and circular component. Origin and destination clusters are sorted in descending order according to traffic volume. The traffic volume within each cluster is shown with bar charts. The temporal axis is preserved for both linear and circular representations. Travel time can be identified in the linear axis.

Commuting efficiency has been explored by Dewulf et al. using floating car and simulated travel demand [79]. The authors used map-based choroplets to visualize average time differences in commuting time during peak and off-peak hours.

**Figure 20:**
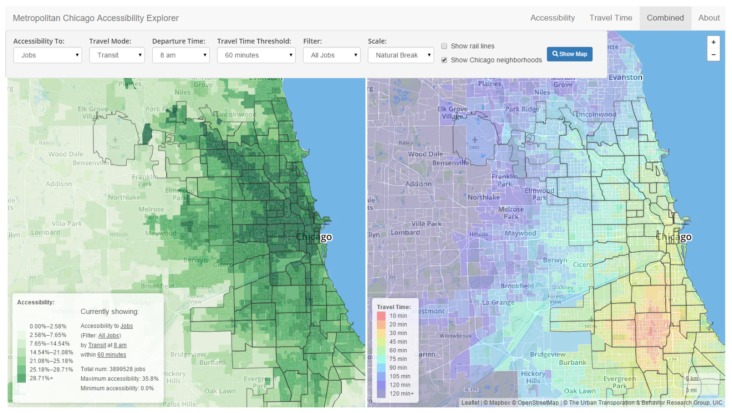
Use of geographic choroplets and heat maps to represent accessibility indexes and travel times [80] (extracted from [82]).

**Figure 21:**
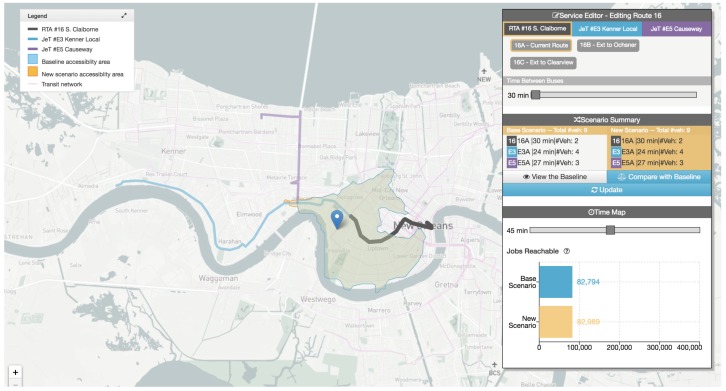
The CoAXs visualization tool for accessibility-based stakeholder engagement [81] (extracted from [83]).

Visualization accessibility measures have been identified in the works of Yin et al., and Stewart and Zegras [80,81]. Both works aimed to identify what activities could be reached by city residents within a given timespan, and spatial (in)equities in terms of transportation availability. Data sources included land use data and transit data such as GTFS schedule data. Map-based visualizations were used to represent isochrones. Yin et al. used heat maps to represent travel time and choroplets to represent accessibility indexes [80], as shown in Figure 20. Stewart et al. proposed used polygon-based isochrones combined with bar charts to show access to job opportunities [81]. The online interactive tool, CoAXs, allows stakeholders to compare two distinct transportation network scenarios (see Figure 21). It is possible to change route parameters, e.g., trajectory, headway and number of buses. In both works, it was possible to identify extensive use of stakeholders on evaluation studies, as both tools are concerned on engaging them on discussion about accessibility.

**Figure 22:**
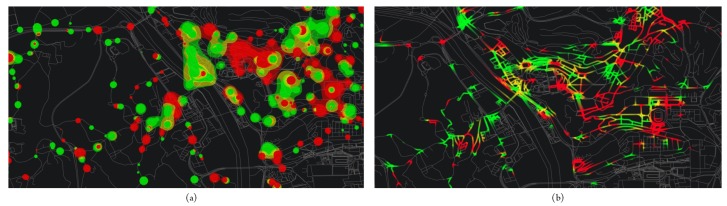
Point (**a**) and vertex-based (**b**) metaballs visualization technique for detecting anomalies on the bus stops of the city of Coimbra, Portugal [84] (extracted from [86]). Colors are related to the sign of the deviation of average number of passengers, with red and green representing above- and below-average deviations, respectively. Colors are related to the sign of the deviation of average number of passengers, with red and green representing above- and below-average deviations, respectively.

Visualization of PTS ridership has been explored in two studies. Data sources included non-APC passenger counts [84] and smart card data from AFC systems [85]. Polisciuc et al. [84] implemented the metaballs visualization technique for the analysis of anomalies on the number of passengers on bus stops, i.e., with significant deviations from the average number of passengers throughout the day. Metaballs were implemented in two ways, as shown in Figure 22: point-based metaballs provide a clear, although exaggerated view of stops with anomalies. Vertex-based metaballs preserves visibility of road network segments, and still allows the identification of areas in which such anomalies occur.

Du et al. used a combination of map-based and abstract technique for analyzing ridership [85]. An abstract calendar visualization was used to show ridership levels for each stop throughout a year. Brushing and linking interactions allow the user to select a specific day and visualize bar charts that show hourly information about ridership for a day.

**Figure 23:**
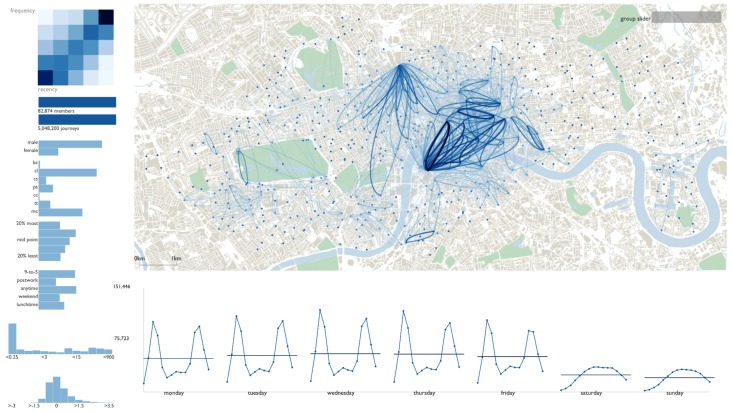
A visual analytics system for exploring cyclists’ trip patterns in London [87].

Cyclists’ trip patterns can also reveal mobility dynamics. The topic was studied by Beecham et al. and Romanillos [87,88]. Wood et al. proposed a visual analytics system (see Figure 23) featuring a combination of coordinated map-based visualization of movements, bar and line plots to represent temporal and thematic information of trips, e.g., trip type, cyclist gender, etc. [87]. Romanillos proposed an online visualization platform for visualization of bicycle routes [88].

The problem of visualizing sparse trajectory data has been addressed by Wang et al. and Chen et al. [14,89]. The latter has already been addressed in the topic People Dynamics in Urban Environments. Wang et al. proposed a visual analytics system based on video stream and vehicle sensors data from ANPR systems [89]. A map-based visualization provides the location of vehicle sensors and road links.

**Figure 24:**
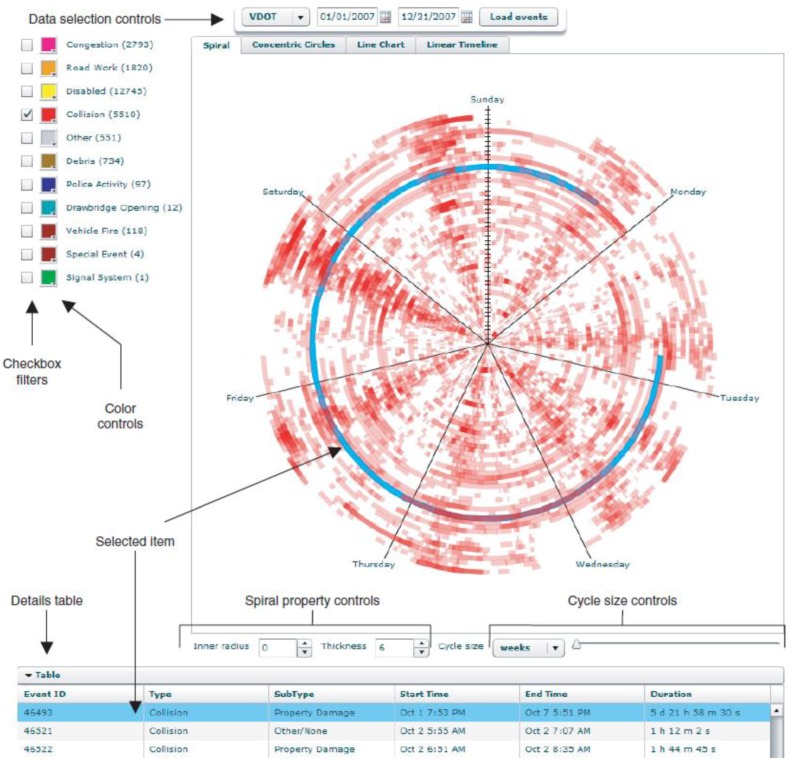
The Spiral Graphs tool for temporal transportation data [90].

VanDaniker proposed the abstract Spiral Graph visualization for temporal transportation data [90]. The visualization tries to overcome the limitations of representing time on linear axes, such as scatter and line plots. Data is plotted on a circular temporal axis, which spirals outward at regular intervals. Figure 24 shows a prototypical visualization tool for collision data. It is possible to visualize the duration of a specific event throughout the circular axis.

Wu et al. proposed a prototypical visualization tool for exploring conversations about traffic using microblogging data [91], with focus on sentiment analysis and trending topics. The system provides abstract visualization techniques mostly based on variations of word clouds.

**Figure 25:**
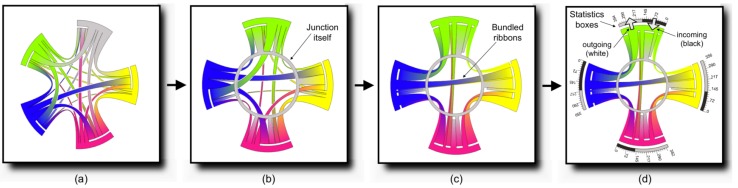
The interchange Circos diagram for visualizing interchange patterns in juction nodes [92]. In (**a**), the original form of the Circos diagram is featured. The improved visualization in (**d**) is obtained by representing junction nodes (**b**) and bundling ribbons (**c**).

Zeng et al. proposed a change to the chord diagrams technique, to visualize interchange patterns in junction nodes, in order to reduce visual clutter (see Figure 25) [92]. Smart card data from AFC systems was used to support visualization development. Frame (a) depicts the original version of the Circos diagram. The junction node is represented as a ring on frame (b), and ribbons are bundled on frame (c), thus reducing visual clutter. Finally, additional statistics such as outgoing and incoming flow are added to facilitate analysis.

Krüger et al. proposed an interactive visualization system, TrajectoryLens for exploring long-term trajectory data [93]. The following interaction tasks are available: focus plus context, dynamic queries and filtering. The system provides multiple coordinate visualization techniques, including a map-based visualization of trajectories, which can be filtered and aggregated by the user. Hierarchical time sliders allow users to filter trajectories according to the desired timespan.

**Figure 26:**
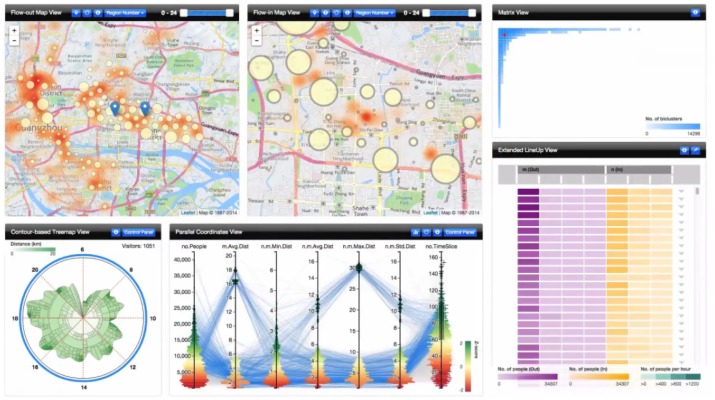
The TelCoVis visualization system for exploring co-occurrence [94] (extracted from [95]).

Wu et al. proposed the TelCoVis visual analytics system for analyzing co-occurrence, i.e., when individuals from two regions visit an urban place during the same timespan. The authors used mobile phone data for supporting the analysis. Similar to other works shown in this section, the system presents multiple coordinated visualization techniques, as shown in Figure 26. Two maps are used to display heat maps for analysis of incoming and outgoing mobility flows (a,b). Abstract visualizations such as matrix heat maps (c,f), contour-based tree map (d) and parallel coordinate plots (e) provide additional information about clusters and their correlations.

## 3. Conclusions

In this article, we provided a survey of literature on the applications of data visualization to urban mobility analysis. The wide availability of data from ITS and advanced visualization frameworks have yielded innovative tools for researchers and practitioners. This review introduced a complementary perspective to existing surveys, which focused on data analysis techniques and broadly categorized movement and general transportation data. We provided a narrower classification of topics on urban mobility (see Table 1), and the types of sensors data from ITS which supported the surveyed studies (see Table 2). In Table 1, topics were sorted in descending order, according to the number of related studies. Table 2 extends the classification proposed by Chen et al. [3] by defining the sensors data types from ITS, their subgroups, as described in Section 1, and related studies. In addition, the group ’Others’ encompasses other types of data sources that are not directly related to ITS and seem to be less exploited in studies: surveys, reports, social networks and mathematical models.

Most studies analyzed urban road traffic and people dynamics, which might be partly explained by the ubiquity of device-based data sources, e.g., GPS-enabled devices, AVL systems and vehicle sensors. In fact, such data sources were frequently exploited by surveyed studies, as shown in Table 2. The proposed visualization techniques aimed at exploring phenomena like traffic congestion and unusual events, e.g., road accidents. Mobile phone data was used for detecting urban hotspots, i.e., places with higher concentration of people. Variations of heat maps, line plots and space-time cubes were used to explore the spatial and temporal perspectives of those phenomena. Some studies proposed dashboards which combined both perspectives through brushing and linking interaction mechanisms, and introduced new visual metaphors to explore other data attributes. It was possible to identify new visual metaphors, e.g., Traffic Origins, for analyzing road accidents and their consequences to an urban network [52].

Vehicle sensors and trajectory data have also been exploited for analyzing air pollution. Related studies relied on traditional geographic heat maps. We argue that researchers could explore and evaluate other visual metaphors based on surveyed studies related to other urban mobility topics.

Visualization of public transportation system data has supported the analysis of passengers’ behavior and ridership, and service reliability. Major data sources are AFC and AVL systems. Traditional visualization techniques have been used, such as line plots for representing time series, bar charts for attributes such as ridership frequency, and geographic heat maps for analyzing the spatial distribution of passenger ridership. Innovative visualization techniques have been proposed, e.g., isotime and OD-pair journey view (Figure 14 and Figure 15), and Trips Explorer (TR-EX) (Figure 16).

The remaining surveyed topics provide opportunities for research on visualization. For instance, the expansion of urban centers require understanding the accessibility and commuting efficiency of people to transportation and job opportunities, as explored by [79,80,81]. Urban traffic conversations extracted from geo-referenced social media data can be further exploited, as in [91], to generate insights on the conditions of a transportation network and people’s commuting habits. Further studies could assess the potential of interactive 3D visualization of urban mobility data, as it has been addressed by a few studies [31,54,74,75]. Finally, all surveyed studies summarized in Table 1 are directed at domain experts and their analytical tasks. Researchers and practitioners could also leverage the potential of data visualization to develop tools to aid citizens throughout the phases of their commuting experience, e.g., planning, waiting and traveling.

## Figures and Tables

**Table 1:** Topics of surveyed studies and their representatives.

Topics	Related Studies
Urban traffic flows and monitoring	[8,15,16,17,18,19,21,22,23,24,25,26,27,28,29,30,31,32,33,34,35,36]
People dynamics in urban environments	[14,36,37,38,39,40,41,42,43,44]
Road traffic incidents	[48,49,50,51,52]
Air pollution	[17,53,54,55,56]
Travel behavior on PTS	[58,59,60,61]
Level of Service on PTS	[64,65,66,67]
Trip patterns	[43,69,70,71]
Big city data	[73,74,75]
Travel demand	[76,77,78]
Public tansportation ridership	[84,85]
Sparse trajectory data	[14,89]
Cyclist behavior	[87,88]
Temporal transportation data	[90,93]
Commuting efficiency	[79]
Accessibility	[80,81]
Urban traffic conversations	[91]
Interchange patterns	[92]
Co-occurrence	[94]

**Table 2:** Data types found in surveyed studies and their representatives.

Groups	Subgroups	Data Types	Related Studies
Sensors	Activity-based	Floating car data	[79]
		Mobile phone data	[37,38,39,40,41,42,76,94]
		Smart card data (AFC)	[58,59,60,61,75,85,92]
	Device-based	Bicycle trajectories data	[87,88]
		Bus AVL data	[15,55,64]
		Bus GPS trajectories	[55]
		Vehicle sensor data	[16,18,19,24,26,30,32,36,52,53,73,74,75,90,96]
		Non-APC Passenger count data	[47,84]
		Taxi GPS trajectories	[22,23,27,28,33,35,43,69,70,71,75,77,78]
		Subway AVL data	[67]
		Tram AVL data	[15,65,66]
		Vehicle GPS trajectories	[25,93]
	Location-based	Video stream data (incl. ANPR)	[31,34,56,73,89]
Others	Survey-based	Household survey data	[74]
		Land use data	[80,81]
		Socio–economic data	[37,76]
		Travel diary survey data	[44,47,76,97]
	Report-based	Car incident record data	[48,49,50,51,52,90]
		Transit data	[64]
		Schedule data	[55,67,80,81]
	Social networks	Microblogging data	[14,32,42,91]
	Model-based	Highway traffic flow data	[21]
		Origin-destination matrices (travel demand)	[44,79]
		Urban traffic flow data (network capacity, travel times)	[8,17,29,36,98]
		Road traffic air pollution (emission and dispersion) or heat	[17,53,54]

## Abbreviations

The following abbreviations are used in this manuscript:

AFC	Automatic Fare Counting
ANPR	Automatic Number-plate Recognition
APC	Automatic Passenger Counting
AVL	Automatic Vehicle Location
CoAXs	Collaborative Accessibility-Based Stakeholder Engagement for Public Transportation Planning
D^2^ITS	Data-Driven Intelligent Transportation System
GIS	Geographic Information System
GPS	Global Positioning System
GTFS	General Transit Feed Specification
ICT	Information and Communication Technology
ITS	Intelligent Transportation Systems
PTS	Public Transportation System
VDGIS	Very Dynamic Geographic Information Systems
